# Durability of cell line xenograft resection models to interrogate tumor micro-environment targeting agents

**DOI:** 10.1038/s41598-019-45444-0

**Published:** 2019-06-24

**Authors:** Ian S. Miller, Liam P. Shiels, Emer Conroy, Kate Connor, Patrick Dicker, William M. Gallagher, Norma O’ Donovan, Robert S. Kerbel, John Crown, Annette T. Byrne

**Affiliations:** 1 Department of Physiology and Medical Physics, Centre for Systems Medicine, Royal College of Surgeons in Ireland, 123 St Stephens Green, Dublin 2, Ireland; 2 Conway Institute, University College Dublin, Belfield, Dublin 4 Ireland; 3 Division of Population Health Sciences, Royal College of Surgeons in Ireland, Lower Mercer Street, Dublin 2, Ireland; 4 National Institute for Cellular Biotechnology, Dublin City University, Glasnevin, Dublin 9 Ireland; 5 Sunnybrook Research Institute, University of Toronto, Bayview Avenue, Toronto, ON M4N 3M5 Canada

**Keywords:** Tumour angiogenesis, Breast cancer

## Abstract

Angiogenesis is a key tumor microenvironment (TME) event underpinning tumor growth and metastasis. Nevertheless, the relatively poor performance of anti-angiogenic therapies in clinical trials compared to pre-clinical studies implies that classical subcutaneous xenograft models have limited predictive potential in this setting. To address this issue, we established orthotopic surgical resection models of breast cancer, which replicate the phenotype of clinical post-resection micro-metastasis. To demonstrate the power and precision of these models, we recapitulated the BETH adjuvant trial (NCT00625898) where the addition of bevacizumab (BVZ) to chemotherapy plus trastuzumab (Trast) failed to provide additional benefit. SCID mice were orthotopically implanted with bioluminescent Her2^+^ MDA-MB-231 or HCC1954 cells and tumors resected c.5 weeks later. Following resection, mice were treated with 10 mg/kg Trast +5 mg/kg paclitaxel (PAC) IP once weekly for 6 cycles +/− weekly BVZ (5 mg/kg IP). Metastasis was monitored by imaging. Using these models our data confirms that the addition of the anti-angiogenic antibody BVZ to adjuvant Trast + chemotherapy provides no additional benefit compared with Trast + chemotherapy alone. Previous studies using non-resection subcutaneously engrafted xenografts failed to predict this outcome. Our results provide compelling evidence for the utility of cell line xenograft resection models to predict clinical outcome for TME targeting agents.

## Introduction

In a seminal paper published in 1971, Dr Judah Folkman hypothesized that tumor growth was dependent on the production of new blood vessels or angiogenesis and therefore inhibition of angiogenesis could be of therapeutic benefit^1^. This groundbreaking hypothesis was based on pre-clinical studies utilizing common syngeneic mouse cell lines and showed that tumors established new vessels from the pre-existing vasculature enabling them to grow beyond 2–3 mm in size^1^. Subsequently, and over several decades, an evolved understanding of angiogenic mechanisms has supported the development of anti- angiogenic agents. Therapies targeted against the Vascular Endothelial Growth Factor A (VEGF-A) pathway, are now widely implemented in the clinic across several tumor types^2^. BVZ, a humanized monoclonal antibody which elicits its effect by binding to VEGF and inhibiting receptor binding, was the first angiogenesis inhibitor approved by the FDA^3^.

The use of BVZ in metastatic HER2-negative breast cancer was approved by the EMA in 2007 and FDA in 2008 after the E2100 trial demonstrated a significantly improved progression free survival (PFS 11.8 vs. 5.9 months, HR 0.60, p < 0.001)^4^. However, in 2010, the FDA reversed its decision to approve BVZ in metastatic HER2-negative breast cancer due to reduced PFS benefits when BVZ was assessed in additional phase III trials using diverse chemotherapy backbones, and further evidence of problematic toxicity profiles, which outweighed the enhanced survival benefits^5^. Nevertheless, and despite a general lack of understanding regarding BVZ mechanism of action in micro-metastatic disease, it was hypothesized that BVZ treatment could be of value as an adjunct to chemotherapy in patients with early stage breast cancer, especially in Her2^+^ disease. Supporting pre-clinical xenograft studies indicated that the combination of trastuzumab (Trast, anti-Her2^+^ monoclonal antibody) plus BVZ was synergistic compared with either treatment delivered as a monotherapy^6^. Several clinical trials were therefore initiated in the adjuvant setting with all showing a disappointing lack of efficacy^7^. One such adjuvant trial (NSABP-B44 “BETH”) sought to determine whether a treatment regimen of chemotherapy plus Trast and BVZ would improve invasive disease-free survival (IDFS), compared with a regimen of chemotherapy plus Trast. No additional benefit with the addition of BVZ to patients’ treatment regimen was observed; an IDFS of 92% at 38 months follow up was evident regardless of the treatment protocol (n = 1757 receiving chemotherapy + Trast and n = 1752 receiving chemotherapy + Trast + BVZ). Moreover, there was an associated increase in adverse events in the BVZ arm^8^.

These clinical results, being in stark contrast to the supporting pre-clinical data^6^, highlight the need for better, more accurate pre-clinical models, which can faithfully predict patient outcomes to novel therapeutics targeting the TME and which might be used in the adjuvant setting. Our co-author (RS Kerbel) has pioneered the use of rodent resection models which mimic the clinical course of tumors following surgical excision of the primary tumor^9^. Implementing these models, we undertook to recapitulate the BETH clinical trial, and to thus investigate if we could accurately replicate clinical outcome. We further employed ^18^F- Fluoro-deoxyglucose positron emission tomography (18F-FDG PET) as a treatment response marker.

## Materials and Methods

### Cell line transfection and culture

Human breast cancer cell lines HCC-1954 (naturally HER2 overexpressing RRID:CVCL_1259 from EACC -UK) and MDA-MB 231/LM2-4/H2N (Engineered to overexpress HER2 as previously described^10,11^ were grown in RPMI-1640 (Sigma), supplemented with 10% (v/v) foetal bovine serum (FBS, Sigma) and L-glutamine (Sigma), in 5% CO_2_ at 37 °C. Cell lines were IMPACT II tested for the presence of mouse pathogens and mycoplasma (IDEXX Laboratories Ltd UK). Both cell lines underwent lentiviral transfection to express the luciferase gene. Bioluminescence was confirmed by plating differing concentrations of cells in a black based 96-well plate in triplicate, incubating overnight followed by addition of 30 mg/ml Luciferin (Perkin Elmer). Prior to the commencement of animal studies, we also performed Western Blot and ELISA analyses to assess HER2 and VEGF expression respectively. Western blots confirmed that both cell lines expressed HER2 (Supplementary Figs 1A and 2A). Using an ELISA we also investigated the secretion of VEGF for both cell lines. VEGF concentrations of 474.39 and 632.75 pg/ml were shown to be present in the conditioned media from HCC1954 and MDA-MB-231-LN2-4/H2N respectively (Supplementary Fig. 3).

#### Compliance with Ethical Standards

Animal experiments conformed to guidelines from Directive 2010/63/EU of the European Parliament on the protection of animals used for scientific purposes. Experiments were licensed and approved by the Health Products Regulatory Authority Ireland (HPRA) project authorization number AE18982/P039. Protocols were also reviewed by University College Dublin Animal Research Ethics Committee (AREC). Power calculations were reviewed and approved by the AREC biostatistician. Specifically, study cohort N number was calculated using the formula N = (Z_α_ + Z_β_)^2^ * (2σ^2^/δ^2^). σ (sigma) is the common variance of tumor volume being measured. δ (delta) is difference between the mean tumor volumes. This difference is judged to be different that testing should generate a significant result (δ = (µ_2_ − µ_1_)).Based on data from Kolinsky *et al*.^12^ we determined that δ = 250 mm^3^, σ = 200 and using standard normal distribution tables α = 0.05 and β = 0.8, (Z_α_ + Z_β_)^2^ = 7.9. Therefore a study cohort size of N = [7.9 × 2 × (200)^2^]/(250)^2^ = 10.112 ≈ 10 animals per group was used. Animals (4–6 weeks 20–25 g, female) were purchased from Charles River (Canterbury, UK) and maintained in specific pathogen free environment.

#### Animal studies

HCC-1954-Luc (10 × 10^6^) or MDA-MB-231/lm/2-4.H2N cells (5 × 10^5^) were orthotopically implanted into the right inguinal mammary fat pad (MFP) of severe combined immunodeficient (SCID: CB17/Icr-Prkdc^scid^/IcrIcoCrl) mice. When tumors reached 400 mm^3^ mice were randomized. Resections were performed as previously described^10^. Due to an ethical limitation of the number of injections a single mouse could receive, docetaxel and carboplatin combination (as per the BETH trial) was replaced by single agent paclitaxel (PAC), without loss of chemotherapeutic effect and following discussion with co-author J Crown (medical oncologist). 3 weeks following resection mice were treated with i) PAC (5 mg/kg) + Trast (loading dose 15 mg/kg then 10 mg/kg) [PT], ii) PAC+ Trast + BVZ (5 mg/kg) [PTB], or iii) Vehicle control [PBS] once per week for 6 weeks (N = 10 per group). Doses of BVZ and Trast were chosen based on previously published studies confirming target inhibition at the doses employed^13,14^. Specifically, Yamashita-Kashima *et al*.^14^ describe by Western Blot analysis the reduction of phosphor-HER2 expression in tumour cells taken from tumours treated with Trast for 4 days at the dose used in the current study (10 mg/kg). Moreover, Mabuchi *et al*.^13^ showed that a dose of 5 mg/kg BVZ inhibited VEGF mediated angiogenesis. They utilized a directed *in vivo* angiogenesis assay where human VEGF (hVEGF) was added to angioreactors, and placed in the flank of mice. VEGF-induced new vessel formation was almost completely inhibited by treatment with BVZ at the doses used in this study.

Tumor volume measurements were compared using a linear mixed model, whereby treatment group and time post treatment were fixed events, and group-by-time interactions and mouse were random effects. Measurements were log-transformed prior to analysis to remove skewness in tumor volume data and to improve model fit.

### Bioluminescence imaging (BLI)

BLI was employed to monitor metastatic growth. Mice were followed until tumors reached 15 mm in any give dimension then euthanized and organs harvested. *Ex vivo* BLI was performed to detect metastatic spread. Imaging was performed with an IVIS Spectrum (Perkin Elmer). 15 mins prior to imaging mice were injected IP with 150 mg/kg Luciferin (Perkin Elmer). A 1 sec reference image was then taken with binning set to 4 and F-stop 1. Image analysis was performed using Living Image software (V4.3.1, Perkin Elmer) and average radiance (p/s/cm^2^/sr) was determined.

### 18F-FDG PET

18F-FDG tumor PET CT was performed on a PET LabPET4Triumph system (Trifoil Imaging). Mice were incubated in a heating box for 30 mins prior to being anaesthetised and injected intravenously (IV) with 6.4 MBq (+/−0.7 MBq) ^18^F-FDG Following injection of ^18^F-FDG, the mice remained under anaesthesia for a 1 hour uptake time, with appropriate heating provided. Mice were then transferred to the PET Triumph, and following the CT scan, a 10 min PET scan was taken using the LabPET software (v1.14.0, Trifoil Imaging) using the X-O CT software (V5.2.0.0 Trifoil Imaging Inc.), which was used to provide anatomical information. The PET image was then re-constructed using 3D MLEM reconstruction algorithms with 20 iterations using the LabPET software and the PET image and CT image were ‘co-registered’ and analysed using PMOD (v 3.208 – PMOD technologies) software. CT image was used as a guide to draw volumes of interest on the PET image and SUVmean for the entire tumor and SUVmax were determined. Where no tumor was present, VOIs were drawn on the surface.

### Immunohistochemistry (IHC)

IHC was performed on all tumor tissues at study termination. Tumor tissue was probed for either Ki-67 (1:150 Santa Cruz, cell proliferation marker) or CD-31 (1: 100 BD Biosciences endothelial cell marker) and counterstained with Haematoxylin. In addition, one section from each tumor was stained with haemotoxylin and eosin (H&E) to determine tumor necrosis. Images were acquired on a Zeiss Axiovert microscope at 200X magnification under bright-field conditions. The resultant photomicrographs were analysed using ImageJ (HIN).

## Results

### Analysis of two orthotopic surgical resection models of Her2^+^ breast cancer show no significant difference in tumour re-growth or survival after treatment with either PT or PTB

**Figure 1:**
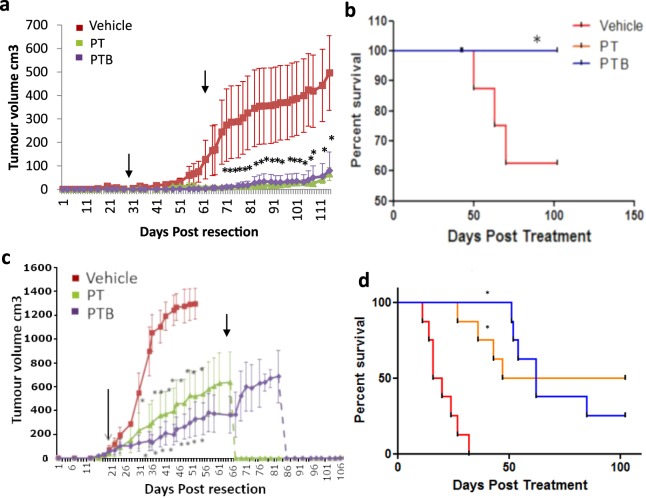
Growth and survival analysis in two orthotopic surgical resection models of Her2^+^ breast cancer show no significant difference in tumor re-growth or survival when comparing adjuvant treatment with PT vs PTB. (**a**) Growth curve showing mean (±SEM) post resection primary tumor re-growth of the HCC 1954 Luc xenograft. 3 weeks following resection all mice were randomly assigned to 3 treatment groups of equal tumor volume, and treatment started. Treatment with Vehicle (▪, n = 8), PT (▴, n = 9), or PTB (♦, n = 9) continued for 6 weeks. Linear mixed model significance test, PT (p = 0.0020) vs PTB group (p = 0.002). No significant difference was observed when PT and PTB cohorts were compared (p = 0.5212). The first arrow shows start of treatment while the second shows completion of treatment. (**b**) Kaplan-Meier survival analysis for the HCC 1954 Luc model. Vehicle n = 8, PT n = 9, PTB n = 9), Censored log rank test; p = 0.032, *on graph. Note that as the survival benefit of treatment with PT and PTB is identical the survival curves are over-layed. (**c**) Growth curve showing post resection primary tumor re-growth in the MDA-MB 231/LM2-4/H2N (Her2+) model. All mice were randomly assigned to 3 treatment groups of equal tumor volume (n = 8 per group) and treatment with Vehicle (▪), PT (▴), or PTB (♦) commenced 3 weeks following resection (First arrow). Treatment was continued for 6 weeks (second arrow shows completion of treatment). A significant difference in tumor volume was observed between the Vehicle group and both the PT (p < 0.0001) and PTB group (p < 0.0001) (Linear mixed model significance test). No significant difference was observed when PT and PTB cohorts were compared (p = 0.9341). (**d**) Kaplan-Meier survival analysis for the MDA-MB 231/LM2-4/H2N (Her2+) model, with 10 week follow up (N = 8 per group). Using a Log-Rank test, a survival benefit was observed for both PT v Vehicle (P = 0.001, * shown on graph) and PTB v Vehicle (p = <0.001, *shown on graph). No significant difference was observed when PT and PTB cohorts were compared.

Both the HCC1954 and MDA-MB-231/LM2-4/H2N models displayed a short latency time following implantation, before quickly reaching 400 mm^3^ at which point they were surgically resected (Fig. 1). Treatment commenced 3 weeks after tumor resection.

#### HCC1954 Model

While there was an 87% primary tumor re-growth rate in the vehicle cohort, treatment groups displayed no re-growth until 3 weeks after treatment cessation, and then at a significantly slower rate than seen in the vehicle cohort. There was a 6/9 recurrence rate of the primary tumour in the PT group and 4/9 in the PTB group (Fig. 1a), however the recurrence rate was not statistically significant (p = 0.6372 Fisher exact test). A significant reduction in mean tumor volume was observed in the PT group (p = 0.002) and the PTB group when compared to the vehicle cohort (Linear mixed model significance test). However, no significant difference in tumor volume was observed between the PT and PTB treatment groups (p = 0.5212). Survival benefits were observed for both treatment groups *vs*. vehicle (Log-rank test, P = 0.032), but no difference was observed between the treatment groups (PT vs PTB) (P = 1.0) (Fig. 1b).

#### MDA-MB-231/lm2-4/H2N Model

Prior to treatment, all animals in each group had evidence of primary tumor regrowth (Fig. 1c). Tumors in the vehicle cohort continued to grow rapidly after the initiation of therapy, with all mice reaching endpoint by day 32 (Fig. 1c). A significant difference in tumor volume was observed between both treatment groups and vehicle (p < 0.0001, Linear mixed model significance test), but not between treatment groups (p = 0.9341) (Fig. 1c). Survival benefits were observed for both treatment groups vs Vehicle (p = 0.001 for both), but not between treatment groups, indicating no additional benefit from the addition of BVZ to the PT combination (P = 0.8260) (Fig. 1d).

#### BLI

**Figure 2:**
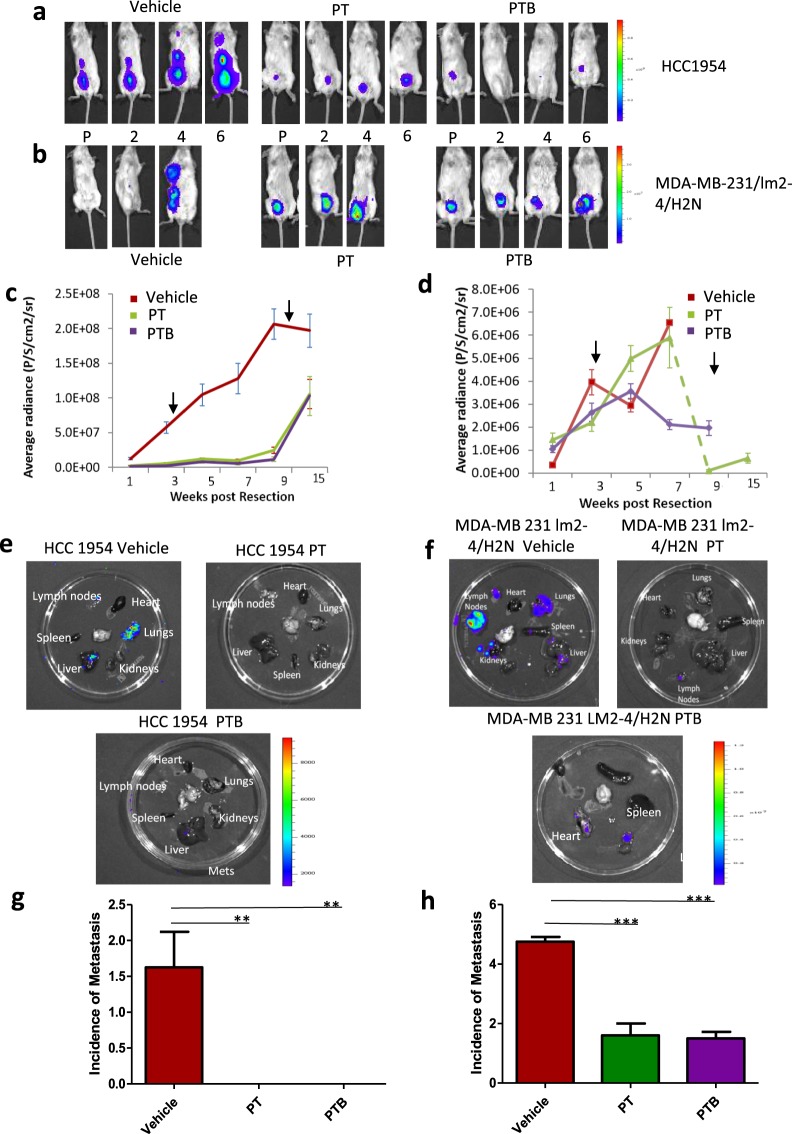
Post resection BLI analysis shows no significant difference in tumor regrowth or metastatic dissemination when comparing response to adjuvant treatment with PT vs PTB in two orthotopic surgical resection models of Her2^+^ breast cancer. BLI data for (**a**) HCC1954 xenografts (Pre-treatment, 2, 4 and 6 weeks post treatment) and (**b**) MDA-MB 231/LM2-4/H2N xenografts (**c**) BLI signal from HCC 1954 xenografts treated with PT or PTB following surgical resection of primary tumors. (**d**) BLI signal from MDA-MB 231/LM2-4/H2N xenografts treated with PT or PTB following surgical resection of primary tumors. Dashed line denotes change in the average radiance (per cohort) due to the death of several animals in the group as a result of tumor regrowth. (**e**) Representative *ex-vivo* bioluminescence in organs taken from HCC1954 tumor bearing mice. Organs from mice treated with either PT or PTB show no evidence of luciferase activity upon *ex-vivo* imaging. Luciferase activity is only evident in the organs of vehicle control animals which metastatic tumors. (**f**) Representative *ex-vivo* bioluminescence in organs taken from MDA-MB 231/LM2-4/H2N tumor bearing mice. Luciferase activity is evident in metastases bearing organs from all treatment groups. However, mice treated with PT and PTB show significantly less luciferase activity compared with vehicle. (**g**) Graph showing total metastases in *ex-vivo* imaged organs of the HCC1954 models as determined by BLI. Neither PT nor PTB treated mice bore metastases as determined by *ex-vivo* BLI. Only mice in the vehicle cohort showed evidence of metastasis (6/8 Vehicle; 0/9 PT and 0/9 PTB), with most metastatic lesions found in the lungs (p = 0.006). (**h**) Graph showing total metastases as determined by BLI in *ex vivo* imaged organs from animals bearing MDA-MB-231/LM2-4/H2N xenografts. Fewer metastases were evident in PT or PTB treated animals compared to the vehicle cohort (Student’s t-test; P = <0.001 v PT and P < 0.001 v PTB (lung metastasis was seen in 8/8 vehicle mice, 3/5 PT mice and 5/6 PTB mice. Distal metastases were seen in 5/8 vehicle mice, 3/5 PT mice and 1/6 PTB mice)). No significant difference was evident in metastatic dissemination when PT and PTB treated animals were compared (Student T-test p = 0.82).

To detect tumor regrowth and subsequent metastatic dissemination after surgical resection of the primary tumor, BLI was employed. In the HCC1954 model BLI signal rapidly increased after primary tumour resection in the vehicle group (Fig. 2a, c), despite the absence of palpable tumors. Another sharp increase in bioluminescence was detected once tumors began to appear. Signal continued to increase until necrosis within the re-growths caused a reduction in bioluminescence. BLI signal increased in treatment groups shortly before the appearance of tumors, 3 weeks post cessation of therapy (Fig. 2c). Implementing *ex vivo* BLI analysis, neither PT nor PTB treated mice showed evidence of metastatic dissemination. Only mice in the vehicle cohort showed evidence of metastasis (6/8 vehicle vs 0/9 PT and 0/9 PTB), with most metastatic lesions found in the lungs (p = 0.006 student T test), (Fig. 2e, g).

For the MDA-MB-231/LM2-4.H2N model, BLI signal increased as tumors grew, with the largest increase in the vehicle group. By treatment cessation all mice in the vehicle group, and all tumor bearing mice in the PT group had reached endpoint. Upon *ex vivo* BLI image analysis (Fig. 2f), fewer metastases were measured in PT or PTB treated animals compared to the vehicle cohort (Student’s t-test; P =  <0.001 v PT and P < 0.001 v PTB (lung metastasis was seen in 8/8 vehicle mice, 3/5 PT mice and 5/6 PTB mice. Distal metastases were seen in 5/8 vehicle mice, 3/5 PT mice and 1/6 PTB mice. No significant difference in metastatic dissemination was evident when PT and PTB treated groups were compared (Student T-test p = 0.82 Fig. 2h).

#### ^18^F-FDG-PET/CT

**Figure 3:**
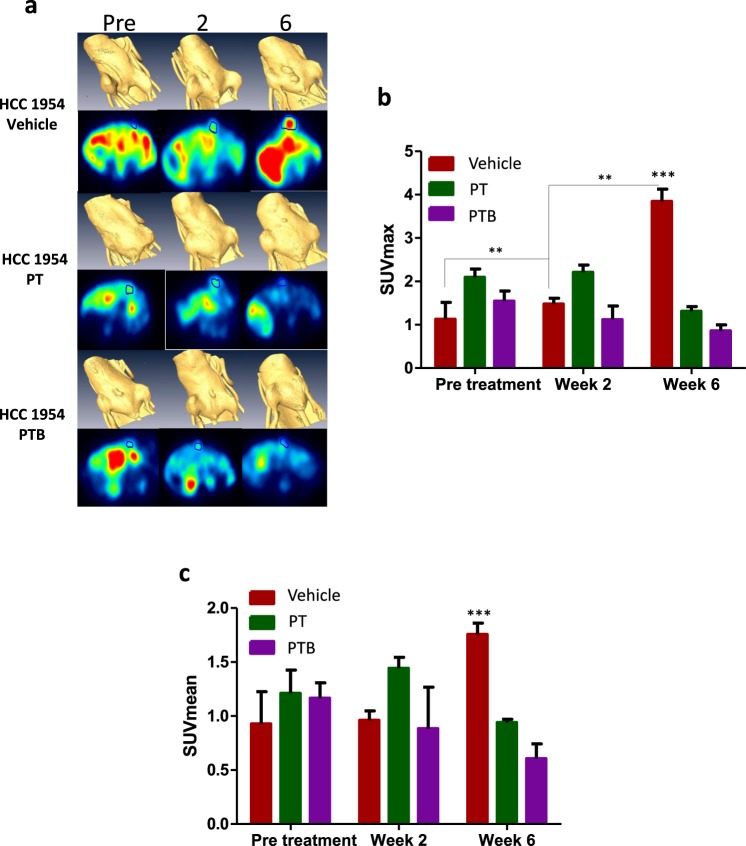
18F-FDG PET analysis of the HCC1954 orthotopic surgical resection model of Her2^+^ breast cancer following PT vs PTB treatment. (**a**) Representative FDG/PET-CT images from HCC 1954 xenograft bearing mice (Pre Treatment, 2 and 6 weeks post-treatment). Blue circle defines area of tumor uptake and measurement. (**b**) Comparison of change in SUVmax over time in HCC 1954 Luc xenograft bearing mice treated with Vehicle, PT, or PTB. A significant increase in SUVmax was observed in the vehicle group between pre-treatment and 6 weeks (ANOVA; p = 0.0048 ** on graph), and between 2 weeks and 6 weeks treatment (ANOVA; p = 0.003 ** on graph). At the 6 week time point there was a significant increase in SUVmax in the vehicle group when compared to either PT or PTB groups (Student T Test; p = 0.0045 and p = 0.0007 respectively, *** on graph). No difference in SUVmax was evident between PT and PTB. (**c**) Comparison of change in SUVmean over time in HCC 1954 xenograft bearing mice treated with Vehicle, PT, or PTB. A significant increase was seen in the SUVmean of the vehicle at 6 weeks of therapy compared to PT or PTB at the 6 week therapy time point (Student T Test; p = 0.0097 and 0.0025 respectively, *** on the graph). No significant difference was determined between SUVmean in PT and PTB treated animals at any time point.

Prior to treatment, n = 3 mice per group from the HCC1954 model were randomly selected to undergo ^18^F-FDG-PET/CT. FDG uptake was assessed as SUVmax (FDG uptake in 5 “hottest” pixels) and as an average uptake (SUVmean). A significant increase in SUVmax over study duration was observed in the vehicle cohort, with increases occurring between pre-treatment and 6 weeks (p = 0.0045) and between 2 weeks and 6 weeks (p = 0.003) (Fig. 3a,b). There was no significant change in SUVmax in the PT treatment group from pre-treatment to 2 weeks post treatment. For the PTB treatment group SUVmax decreased over the course of the study, with the change between pre-treatment and 6 weeks treatment nearing significance (p = 0.0511). Similarly, when analyzing SUVmean there was a significant difference between vehicle treated mice and PT/PTB mice (p = 0.0097 and 0.0025 respectively), yet no significant difference was detected between PT and PTB at either week 2 or week 6 time point.

#### IHC Analysis

**Figure 4:**
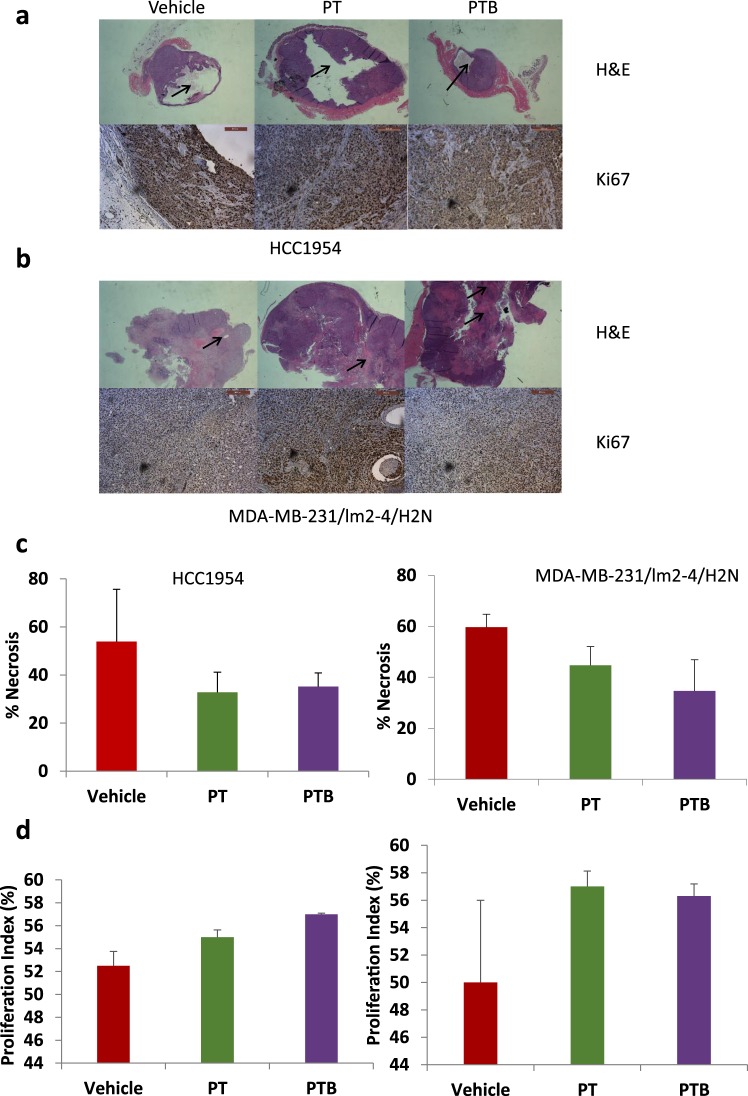
Immunohistochemical analysis of primary tumor regrowth in two orthotopic surgical resection models of Her2^+^ breast cancer show no difference in proliferation or necrosis at 10 weeks following treatment cessation. (**a**) Representative images of H&E stained tissue sections and Ki67 probed HCC1954 tumor sections treated with vehicle, PT and PTB of post resection tumor regrowth from HCC 1954 resected tumors. Tumors were take 10 weeks after therapy cessation. Arrows point to areas of necrosis within tumor. Ki67 positive cells are stained brown while total nuclei are stained blue. (**b**) Representative images of H&E stained tissue sections and KI 67 probed MDA-MB-231 LM2-4 H2N tumor sections treated with vehicle, PT and PTB of post resection tumor regrowth from HCC 1954 resected tumors. Tumors were take 10 weeks after therapy cessation. Arrows point to areas of necrosis within tumor. Ki67 positive cells are stained brown while total nuclei are stained blue. (**c**) Percentage necrosis was measured in both the HCC1954 and MDA MB 231 LM2-4 H2N cell lines at 10 weeks after the cessation of therapy. The area of necrosis was calculated by the ratio between the area of dead tissue: total tumor area. No significant difference (2 tailed student T test) in % necrosis was evident between vehicle, PT or PTB in either model. Error bars represent standard error of the mean. N = 3 per group. (**d**) Levels of Ki-67 expression were quantified as a percentage of total cell population by brown nuclei (Ki 67^+^) vs blue nuclei (total cell number) analysis N = 3 per group. No significant difference was detected between vehicle, PT or PTB treated tumors (Students T-test) in either model after 10 weeks after therapy cessation Error bars represent standard error of the mean.

To investigate tumor necrosis (H&E), proliferation (Ki-67) and micro-vessel density (CD-31) tumors were removed at study endpoint (15 mm diameter), or following a 10-week post treatment follow up period and stained for H&E, Ki-67 or CD-31, and relative protein levels quantified. In both the HCC 1954 (Fig. 4a) and MDA-MB 231/LM2-4/H2N (Fig. 4b) model H&E staining was performed on post resection tumor re-growths to assess tumor necrosis (black arrow heads). While there was a trend towards increased necrosis in the vehicle cohort, no significant difference was observed between any of the groups (Fig. 4c). Similarly, Ki-67 staining (proliferation) displayed no differences between treatment groups (Fig. 4d). A likely explanation is that any direct effects of treatment on cancer hallmarks may no longer be evident in these resistant tumors at time of sacrifice (10 weeks post treatment). CD31 immunostaining was inconclusive; Several sections from each tumor cohort contained no discernible vessels and no clear staining pattern.

## Discussion

Tumor growth and metastasis are highly dependent on new vessel formation. Therefore it has been long understood^1^ that inhibition of angiogenic signalling represents a valid targeted approach for disrupting tumor progression. Pro-angiogenic factors are produced from early stages of tumor growth. Of these factors, VEGF has been the most studied. It acts as a mitogen, survival and migration factor for endothelial cells and is a potent inducer of vascular permeability^15^. Supported by positive preclinical and clinical evidence, BVZ became the first VEGF-targeting drug approved by the FDA and has subsequently been approved for multiple cancer indications^6^. However, while BVZ works in the metastatic setting (for a subset of patients), it has failed to produce a significant effect when delivered in the adjuvant setting^16,17^, despite the availability of positive pre-clinical data^6^. This lack of clinical response is likely due to tumor microenvironment differences between the well-established macro-metastatic lesion and the micro-metastatic niche present in early stage disease^18^. Failure to translate findings from animal studies to humans is in part due to the limited ability of models to accurately mimic the early disease phenotype following surgery.

Nevertheless, phase III trials evaluating the addition of BVZ to the standard chemotherapeutic regimens in the *neoadjuvant* setting (ie prior to surgery) have demonstrated improved responses^19–21^. Specifically, von Minckwitz *et al*. have reported that the addition of BVZ to neoadjuvant chemotherapy significantly increased the rate of pathological complete response (pCR) (14.9% chemo alone vs 18.4% chemo plus BVZ) among patients with HER2-negative early-stage breast cancer, specifically those with early stage node negative triple negative disease^19^. Additional studies have further demonstrated an increase in pCR with the addition of BVZ to neoadjuvant chemotherapy backbones^20,21^. Motivated by these findings, our co-author (RS Kerbel) has implemented clinically relevant surgical resection mouse models, to show the benefit of the antiangiogenic aflibercept (VEGF Trap) when given in the neoadjuvant setting^22^. Specifically, implementing a resectable mouse model of triple negative breast cancer, aflibercept when combined with PAC was shown to further decrease primary tumor burden when compared with single agent PAC treatment.

As the durability and predictive power of resectable xenograft models has previously been shown in the neoadjuvant^22^ and metastatic setting^23^, we employed Her2^+^ breast cancer xenografts to accurately recapitulate the clinical *adjuvant* disease setting. To demonstrate the enduring utility of these xenografts (when used together with a resection protocol) we have recapitulated the negative results seen in the BETH clinical trial where the addition of BVZ to adjuvant Trast + chemotherapy provided no additional benefit. Here, we compared primary tumor regrowth, survival, incidence of metastases and FDG uptake following PT and PTB adjuvant treatment in two resectable Her2 + breast cancer xenografts and have shown that BVZ combined with PAC and Trast (PTB) when delivered post resection provides no additional benefit compared with the PAC+ Trast(PT) combination

When considering treatment response in the resectable Her 2+ models employed, it is important to consider the effect of the PT combination alone. This, to ensure that any benefit due to the addition of BVZ is detectable beyond the effects of PT. In this context and considering the effect of PT in the MDA-MB-231/LM2-4/H2N (HER2^+^) model it is clear that PT treatment has a significant effect on tumor regrowth. Nevertheless, maximal inhibition is not achieved (Fig. 1c PT curve, green line). Moreover, when considering the impact of PT treatment on survival (Fig. 1d PT curve: orange line) it is also clear that PT does not ‘cure’ all animals (ie maximal survival benefit is not reached). Therefore, if a more effective treatment was deployed, additional tumor regrowth or survival benefits could be revealed. Here, we show that the addition of BVZ to the PT regimen does *not* provide any additional benefit when considering tumor regrowth or survival. Specifically, no significant difference was seen when tumor regrowth (P = 0.9341) or survival was compared between PT and PTB cohorts (P = 0.826). In the same model we also see evidence of metastases following treatment in all cohorts (Fig. 2f, h). Our data clearly shows that PT treatment alone does not fully eliminate metastases (green bar Fig. 2h). Indeed, it would certainly be possible to see a further reduction in metastatic burden if a more effective treatment was employed. However, we show that the addition of BVZ to the PT regimen does not provide any additional benefit (ie does not further reduce metastatic burden). Specifically, no significant difference was seen when post-treatment metastatic burden was compared between PT and PTB cohorts (P = 0.82). Nevertheless, when considering response to treatment in the HCC1954 model; PT ostensibly elicts a maximal effect on tumor regrowth (Fig. 1a), survival (Fig. 1b) and metastatic burden (Fig. 2e, g). Considering these three response criteria alone it is difficult to show added benefit of any additional intervention beyond that which is evident in the PT cohort. However, by considering the highly sensitive FDG-PET imaging data presented in Fig. 3b,c, it is clear that FDG uptake at 2 weeks and 6 weeks is not maximally inhibited by PT (Fig. 3b Week 6 green bar; Fig. 3c Week 6 green bar). Thus, it would certainly be possible to see a further reduction in tracer uptake if a more effective treatment was employed. Here, we show that the addition of BVZ to the PT regimen does not provide any additional benefit (ie does not further reduce FDG uptake at Week 2 or Week 6). Specifically, no significant difference was seen when post-treatment (Week 2 and Week 6) SUVmax or SUVmean was compared between PT and PTB cohorts (P = 0.2281 and P = 0.2244 respectively).

In short, the pre-clinical findings presented here are in stark contrast to the pre-clinical data which supported the BETH trial. In these earlier studies which employed HER2-overexpressing human subcutaneous breast cancer xenografts, combined treatment with Trast plus BVZ resulted in significant reduction in tumor volume compared to Trast alone^6^. Clearly SQ models only support the assessment of treatment effects on primary tumor growth and are inappropriate for assessing adjuvant TME-targeting therapeutics.

A further point of note is the observation both here (with BVZ) and also previously^24^ that anti-angiogenic treatment (with sunitinib) of the primary tumor showed no evidence of overt tumor regression but instead provides evidence of growth delay. In the clinic, such a growth delay would be viewed as treatment failure or stable disease at best. Nothing close to a pathologic complete response or even partial response was observed in either study. In the future, it will be of interest to see if a therapy, which causes an effect resembling a pathologic complete response in a primary tumor model, would show efficacy in the post-surgical adjuvant or overt metastatic setting, and hence predict metastatic efficacy. Admittedly however, there is currently a dearth of drugs that would currently elicit such a response in an established mouse tumor.

The enduring problem of misleading pre-clinical findings results in thousands of patients undergoing treatments with ineffective and potentially toxic therapies, and billions of wasted healthcare dollars. Considering the negative outcomes of the BETH, BEATRICE and E2100 adjuvant breast cancer trials alone, >5,500 women were enrolled^4,8,25^ at an average cost of ~$67000 per patient^26^. Thus, there continues to exist an urgent need for more accurate and clinically relevant animal models which better mimic the human disease phenotype and its progression, and which are particularly suited to the accurate prediction of clinical outcomes for adjuvant TME targeting therapies.

Here we have shown that implementation of clinically relevant micro-metastatic models which incorporate surgical resection protocols are appropriate for assessing adjuvant anti-angiogenic regimens. We have further demonstrated that it is possible to accurately predict clinical treatment outcomes with cell line xenografts. We thus provide a convincing argument that cell line xenograft models continue to hold significant value for screening of antiangiogenics (and likely other TME targeting agents) even within the prevailing Patient Derived Xenograft/GEMM era. In particular, while we^27,28^ have recently discussed how the cellular, molecular, and genetic properties of PDXs better recapitulate patient tumors compared to established cell lines grown in tissue culture for many passages, PDX models are (i) generally implanted subcutaneously (ii) are rarely resected and (iii) rarely metastasize. Therefore, it is as yet unclear if PDX population studies can reliably predict outcomes of new treatment regimens designed for use in the adjuvant setting. As GEMMs frequently present with multiple primary tumors which arise asynchronously over time, surgical resection experiments are either highly impractical or impossible in these models.

In conclusion, when used appropriately, we assert that our data supports a continued and important role for cell line xenograft models when interrogating novel TME targeting adjuvant therapies in the pre-clinical setting.

## Supplementary information

Supplementary Information

## Data Availability

All data generated or analysed within this study are included in this published article (and its Supplementary Information files).
