# Supplementary material for: Durability of cell line xenograft resection models to interrogate tumor micro-environment targeting agents
Source: Sci Rep. 2019 Jun 24;9:9204. doi: 10.1038/s41598-019-45444-0 (PMC6591410; doi:10.1038/s41598-019-45444-0)
Supplement: Supplementary file 1 — Supplementary Information [file 41598_2019_45444_MOESM1_ESM.pdf]

Supplementary Information

Durability of cell line xenograft resection models to interrogate tumor micro-environment targeting agents.

Ian S. Miller[1$], Liam P. Shiels[1$], Emer Conroy[2], Kate Connor[1], Pat Dicker[3] William M. Gallagher[2], Norma O'Donovan[4], Robert S. Kerbel[5], John Crown[3], Annette T. Byrne[1,2*]

[1]Department of Physiology and Medical Physics, Centre for Systems Medicine, Royal College of Surgeons in Ireland, 123 St Stephens Green, Dublin 2, Ireland; [2]Conway Institute, University College Dublin, Belfield, Dublin 4, Ireland; [3]Division of Population Health Sciences, Royal College of Surgeons in Ireland, Lower Mercer Street, Dublin 2, Ireland; [4]National Institute for Cellular Biotechnology, Dublin City University, Glasnevin, Dublin 9, Ireland, [5]Sunnybrook Research Institute, University of Toronto, Canada;

a

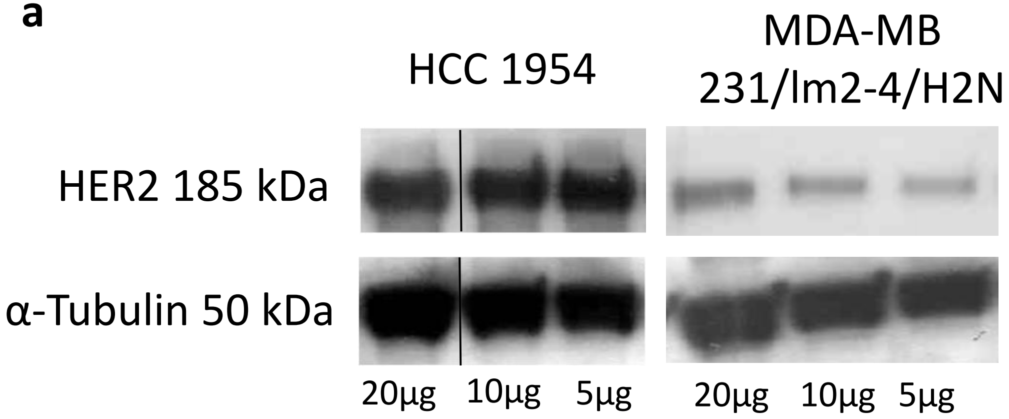

b

Sample	Mean Optical Density	Mean Concentration (pg/ml)
MDA-MB 231/lm2-4/H2N	0.977	474.4
HCC 1954	1.242	632.8

Supplementary Figure 1: Confirmation of HER2 expression and VEGF secretion in HCC 1954 and MDA-MB 231/lm2-4/H2N cell lines

(A) Western blot analyses of 5-20µg whole cell protein of both MDA-MB-231/lm2-4/H2N and HCC1954. Membranes were probed for the presence of the HER2. α tubulin was used to ensure equal protein loading between cell line. The presence of a band at 185kDa confirmed the over-expression of HER2 in both cell lines. Blot for HCC 1954 cell line has been cropped to remove lane containing overspill from previous sample. Full-length blots are displayed separately in Supplementary figure 2. (B) Table showing quantification of VEGF secretion for both cell lines as determined by ELISA assay. Absorbance values (@ 405nm) for the samples were compared to a standard curve to determine the amount of VEGF in each sample. Each sample was analysed in triplicate.

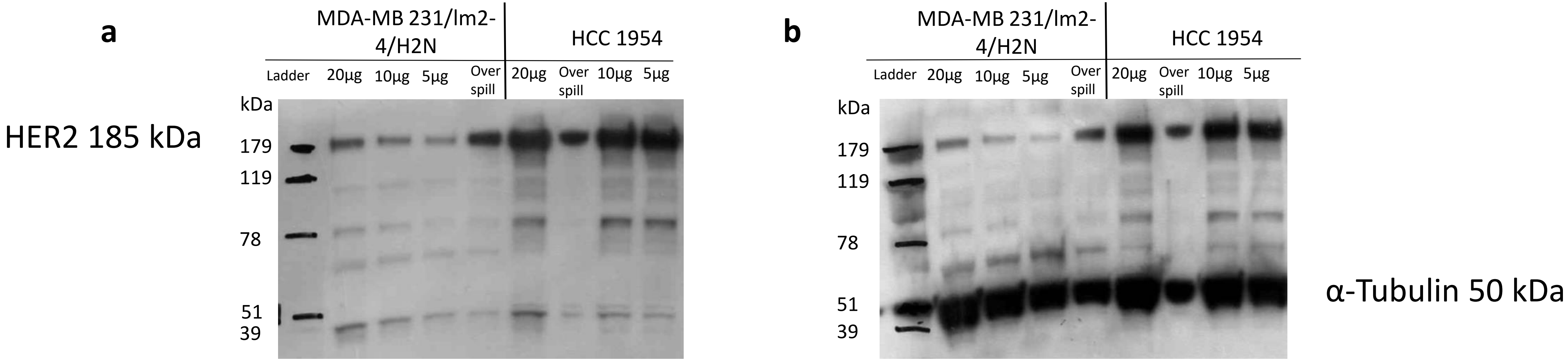

Supplementary Figure 2: Confirmation of HER2 expression in HCC 1954 and MDA-MB 231/lm2-4/H2N cell lines

(A) Western blot analyses of 5-20µg whole cell protein of both MDA-MB-231/lm2-4/H2N and HCC1954. Membranes were probed for the presence of the HER2. The presence of a band at 185kDa confirmed the over-expression of HER2 in both cell lines. (B) HER2 blot was subsequently stripped to remove the HER2 antibody and was then re-probed with α-tubulin. α-tubulin was used as a loading control to ensure equal protein loading between cell lines.

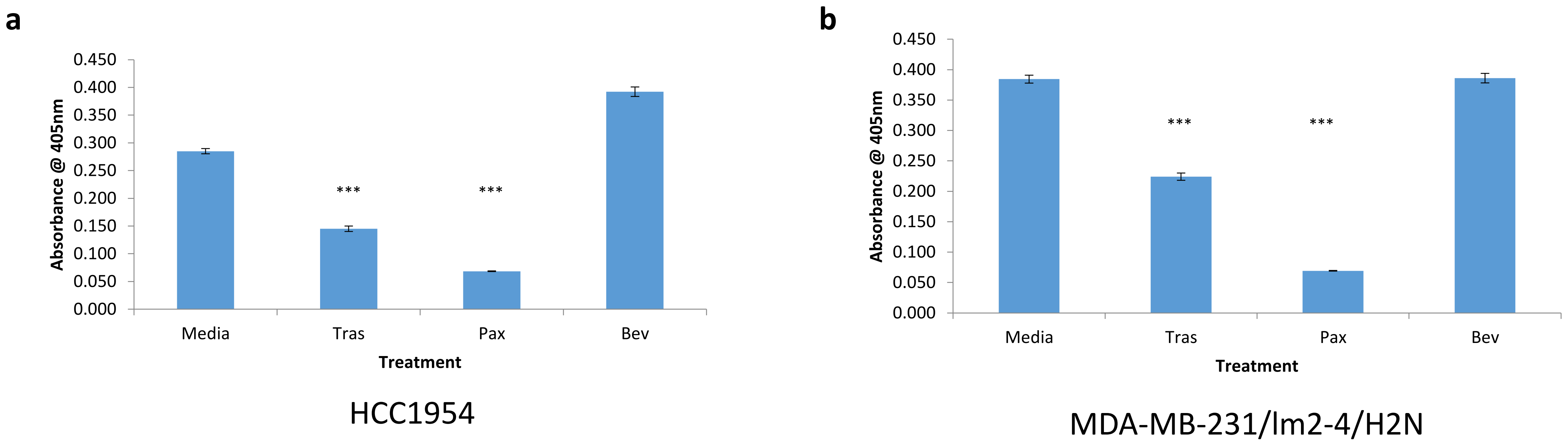

Supplementary Figure 3: In vitro assessment of response to Trast, PAC and BVZ in HCC 1954 and MDA-MB 231/lm2-4/H2N cell lines

(A) Graph showing the survival (by acid phosphatase assay) of HCC 1954 cells following 72 hours treatment with Trast (10 µg/mL), PAC(20 µg/mL) or BVZ(10 µg/mL). Both Trast and PAC significantly decreased cell survival of HCC1954 cells as measured by acid phosphatase (Absorbance @405nm P<0.001 for trastuzumab and paclitaxel n=20). Bevacizumab (10µg/mL) did not a cause a significant difference in cellular survival. (B) Graph showing the survival of MDA-MB-231/lm2-4/H2N cells following 72 hours treatment with Trast (10 µg/ml), PAC (20 µg/ml) or BVZ (10 µg/ml). As with the HCC1954 cell line, both Trast and PAC significantly decreased cell survival of MDA-MB-231/lm2-4/H2N as measured by acid phosphatase (absorbance @405nm P<0.001 for trastuzumab and paclitaxel n=20). As expected, treatment with BVZ (10µg/mL) did not cause a significant decrease in tumor cell survival in vitro and in the absence of supporting blood vessels.
